# Identification of the RP11-21C4.1/SVEP1 gene pair associated with FAT2 mutations as a potential biomarker in gastric cancer

**DOI:** 10.1080/21655979.2021.1953211

**Published:** 2021-07-24

**Authors:** Lingshan Zhou, Yuan Yang, Min Liu, Yuling Gan, Rong Liu, Man Ren, Ya Zheng, Yuping Wang, Yongning Zhou

**Affiliations:** a Department of Gastroenterology, The First Hospital of Lanzhou University, Lanzhou, China; b Department of Geriatrics Ward 2, The First Hospital of Lanzhou University, Lanzhou, China; c Department 1nd Department of Bone and Soft Tissue Oncology, Gansu Provincial Cancer Hospital, Lanzhou, China

**Keywords:** RP11-21C4.1, SVEP1, prognosis, gastric cancer

## Abstract

Gastric cancer (GC) is one of the most common malignancies worldwide. Despite rapid advances in systemic therapy, GC remains the third leading cause of cancer-related deaths. We aimed to identify a novel prognostic signature associated with FAT2 mutations in GC. We analyzed the expression levels of FAT2-mutant and FAT2-wildtype GC samples obtained from The Cancer Genome Atlas (TCGA). The Kaplan–Meier survival curve showed that patients with FAT2 mutations showed better prognosis than those without the mutation. Sixteen long non-coding RNAs (lncRNAs) and 62 messenger RNAs (mRNAs) associated with FAT2 mutations were correlated with the prognosis of GC. We then constructed a 4-mRNA signature and a 5-lncRNA signature for GC. Finally, we identified the most relevant *RP11-21 C4.1/SVEP1* gene pair as a prognostic signature of GC that exhibited superior predictive performance in comparison with the 4-mRNA or 5-lncRNA signature by weighted gene correlation network analysis (WGCNA) and Cox proportional hazards regression analysis. In this study, we constructed a prognostic signature of GC by integrative genomics analysis, which also provided insights into the molecular mechanisms linked to FAT2 mutations in GC.

## Graphical abstract

**Figure:**
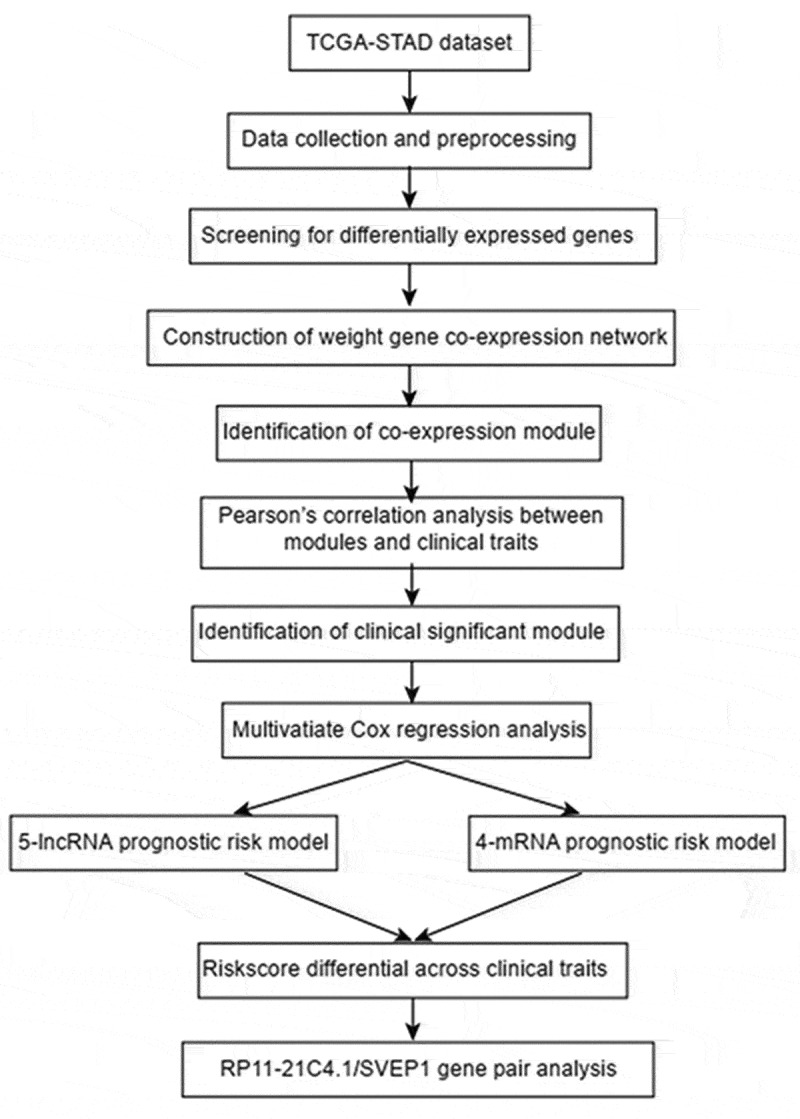

## Introduction

Gastric cancer (GC) is the fifth-most common malignancy worldwide. Despite the global decline in the incidence and mortality rates of this cancer since the last mid-century, GC remains the third leading cause of cancer-related deaths, with an estimated 783,000 deaths in 2018 [1]. Although surgery is regarded as the principal treatment with curative intent, 40%–60% of those who undergo resection surgery show disease relapse [2]. The prognosis of these patients is poor, with a 5-year survival rate of less than 10% [1]. Various genetic mutations have been found to play vital roles in the development of cancer and are considered potential hallmarks of the disease [3]. Alterations in the expression patterns of these genes can lead to different clinical outcomes. With the advancements in genomics, the clinical classification of cancer has begun to shift from a histology-based to a biomarker-driven process [4]. Thus, the search for prognostic biomarkers is a promising and essential field of study [5].

FAT atypical cadherin 2 (*FAT2*) is a homolog of the *Drosophil*a *fat* gene, which is involved in Hippo signaling cascades and tumor suppression [6]. Mutations in *FAT2* frequently occur in many types of tumors, such as spinal meningiomas, malignant mesotheliomas, esophageal squamous cell carcinoma, and colorectal cancer [7–10]. *FAT2* knockdown inhibits the migration of human squamous carcinoma cells and induction of FAT2 by ΔNp63α promotes tumor invasion, indicating that alterations in FAT2 expression play a role in the development of tumors [11,12]. However, studies on FAT2 in gastric cancer are relatively scarce. Only one study has shown that FAT2 is significantly correlated with lymph node and distant metastases and poor prognosis in GC [13]. However, the effect of FAT2 mutations on survival and the mechanism by which FAT2 mutations affect tumor progression in GC remain unclear and require elucidation.

The present study aimed to explore the correlation between FAT2 mutations and the prognosis of GC, as well as the molecular mechanisms linked to FAT2 mutations in GC by combining genetic mutation, mRNA expression, and lncRNA expression information obtained from TCGA, in order to identify a novel prognostic biomarker for GC based on FAT2 mutations. In comparison with studies evaluating the molecular levels separately, integrative genomics analysis yields a higher information content [14]. We found that FAT2 mutations were associated with a favorable prognosis in GC. Lastly, a transcriptomic gene pair was identified as a prognostic biomarker for GC by analyzing the differentially expressed mRNAs (DEmRNAs) and differentially expressed lncRNAs (DElncRNAs) associated with FAT2 mutations.

## Materials and methods

### Data sources

The somatic mutation, transcriptome, and corresponding clinical data of stomach adenocarcinoma (STAD) were downloaded from TCGA (https://portal.gdc.cancer.gov). Patients with GC were classified into FAT2-mutant and FAT2-wildtype groups.

### Survival analysis

The Kaplan–Meier method was used to generate survival curves for the FAT2-mutant and FAT2-wildtype groups. The differences in survival curves were determined using the log-rank test. Statistical analyses were performed with R. A P-value of <0.05 was regarded as statistically significant.

### Differentially expressed gene (DEG) analysis and functional enrichment analysis

The R package ‘edgeR’ was used to identify the DElncRNAs and DEmRNAs between FAT2-mutant and FAT2-wildtype groups with a fold-change analysis and t-test. Genes with an absolute fold change (log_2_) of >2 and adjusted P-value of <0.05 were considered as DEGs. To explore the biological attributes of these DEGs, we conducted Gene Ontology (GO) analysis and Kyoto Encyclopedia of Genes and Genomes (KEGG) analysis using the R package ‘clusterProfiler.’ Gene-set enrichment analysis (GSEA) was used to identify statistically significant sets of genes based on candidate genes.

### Weighted gene co-expression network analysis (WGCNA)

WGCNA was performed with the R package ‘WGCNA’ to create and analyze a co-expression network for DEGs. The STAD samples were clustered using average linkage and Pearson’s correlation coefficients. A power of β equal to 4 was selected as the soft-threshold parameter to construct the co-expression gene network. Then, we calculated a topological overlap matrix (TOM) using the adjacency matrix. TOM dissimilarity was evaluated for module partition analysis. A hierarchical clustering tree of genes was constructed to classify genes with similar expression patterns. Subsequently, we used the Dynamic Tree Cut algorithm to obtain the modules of the network by cutting the branches of the tree. Finally, the most important genes in each selected module were used for further analysis. This step was based on two limiting parameters: gene significance (GS) and module membership (MM).

### Construction of lncRNA and mRNA prognostic risk models

The genes in the blue-green module were subjected to univariate Cox proportional hazards regression analysis. Genes associated with prognosis were also identified. Subsequently, we used the least absolute shrinkage and selection operator (LASSO) analysis to filter genes. Cox regression analysis was performed to develop prognostic risk signatures with the screened genes. The risk score of each patient was calculated using the following formula: risk score=$\sum_{i=1}^{n}$. (expression level of gene * regression coefficient). On the basis of the median risk score, patients with GC were divided into high- and low-risk groups, and Kaplan–Meier survival curves were constructed to compare the OS of the two groups. The R package ‘survivalROC’ was applied to assess the predictive potential of the two models by measuring their AUC values.

## *Identification and analysis of the* RP11-21C4.1/SVEP1 *gene pair*

We explored the correlation between mRNAs and lncRNAs in the prognostic models. The most relevant gene pair was identified using Pearson correlation analysis. The rank sum test was used to compare DElncRNAs and DEmRNAs. The association between *RP11-21C4.1/SVEP1* and clinical characteristics was analyzed using logistic regression.

### Construction of the TF-mRNA network on the basis of the candidate genes

We used the R package ‘limma’ to identify DEGs between the SVEP1 high-expression and low-expression groups. Then, we used the module ‘UCSC_TFBS’ under the ‘Protein Interactions’ function of DAVID to annotate the DEG list. Finally, the identified TFs were visualized using Cytoscape software.

## Results

This study aimed to construct a gene pair prognostic signature in GC associated with FAT2 mutations. We explored the correlation between FAT2 mutations and the prognosis of GC by using the Kaplan–Meier method, and further analyzed the differentially expressed mRNAs and lncRNAs based on the FAT2 mutation status. The prognostic mRNAs and lncRNAs were identified, and an lncRNA/mRNA gene pair prognostic signature was constructed by weighted gene correlation network analysis (WGCNA) and Cox proportional hazards regression analysis. Our study yielded a precise predictor of the prognosis of patients with GC and could provide support for clinical decision-making.

### The prognosis of FAT2 mutations in GC

**Figure 1.**
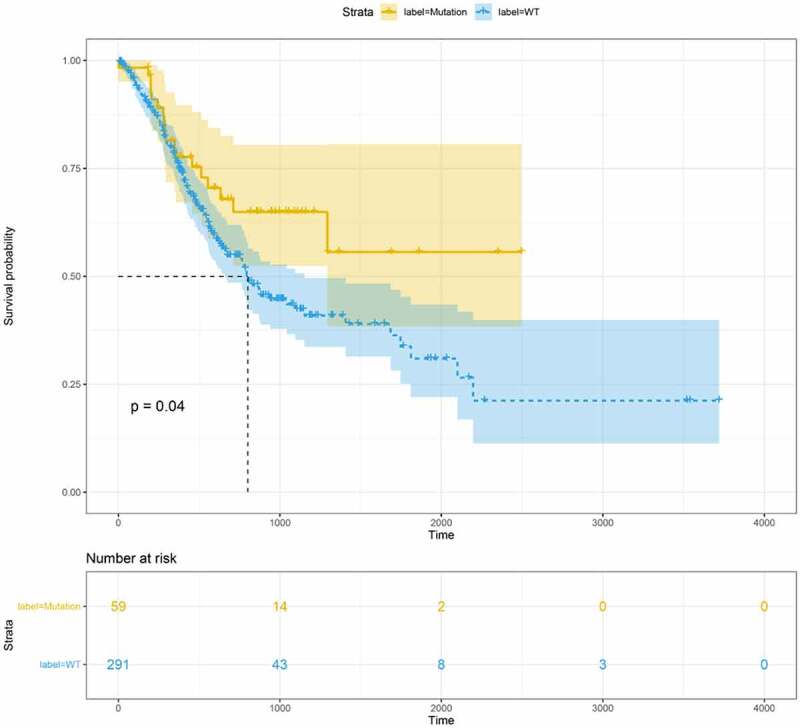
Overall survival of GC patients in the FAT2-mutant and FAT2-wildtype groups

A total of 375 STAD samples obtained from TCGA were divided into the FAT2-mutant (n = 63) and the FAT2-wildtype (n = 312) groups. After excluding the samples without clinical data, 59 FAT2-mutant and 291 FAT2-wildtype samples were subjected to survival analysis. The FAT2-mutant frequency in GC was approximately 16.8%. The FAT2 mutations in patients with GC were mainly missense, truncating, and splice mutations, with missense mutations being the most common. We further validated the FAT2 mutations in another database obtained from cbioportal, which showed a 14% mutant frequency in GC. The KM survival curve was drawn to explore the effect of FAT2 mutations on the prognosis of GC. In comparison with patients without FAT2 mutations, those with FAT2 mutations showed a better prognosis (Figure 1).

### Identification of the DElncRNAs and DEmRNAs between FAT2-mutant and FAT2-wildtype samples of GC

**Figure 2.**
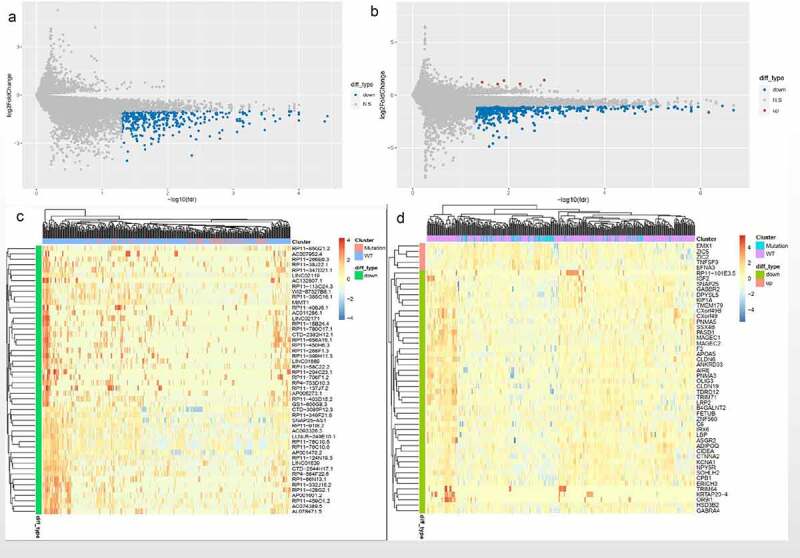
Identification of the DElncRNAs and DEmRNAs between the FAT2-mutant and FAT2-wildtype groups. (a) Volcano plot for DElncRNAs. (b) Volcano plot for DEmRNAs. (c) Heatmaps for the DElncRNAs. (d) Heatmaps for the DEmRNAs

**Figure 3.**
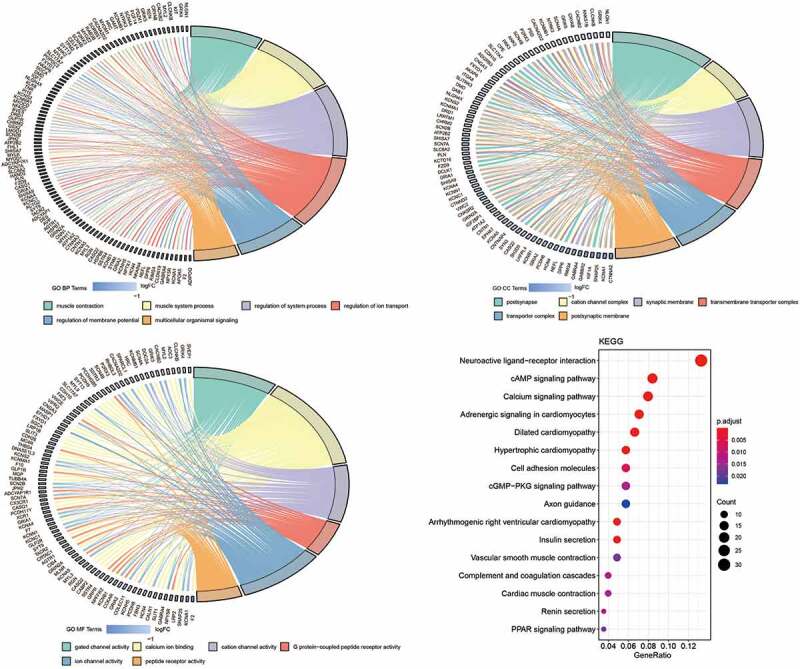
Functional enrichment analysis related to DEmRNAs. (a) Significantly enriched biological processes in the GO analysis. (b) Significantly enriched cell components in the GO analysis. (c) Significantly enriched molecular function in the GO analysis. (d) Significantly enriched pathways in KEGG analysis

To explore the potential mechanism of FAT2 mutations in GC, we identified the transcriptomic features associated with FAT2 mutations. A total of 365 DElncRNAs were downregulated in FAT2-mutant samples. Meanwhile, 576 DEmRNAs were identified, of which 5 and 571 were upregulated and downregulated, respectively (Figure 2(a-d)). Then, we performed GO and KEGG analyses to explore the characteristic biological attributes of these DEmRNAs. Our results showed that the DEmRNAs were mainly enriched in the intracellular material transport and signal transduction-related biological processes and pathways, such as regulation of ion transport, regulation of membrane potential, multicellular organismal signaling, transporter complex, gated channel activity, cAMP signaling pathway, calcium signaling pathway, and cell adhesion molecules (Figure 3(a-d)). These findings demonstrated that the identified DEmRNAs played an important role in biological function, and these related processes and pathways might underlie the effects of FAT2 mutations in the development of GC.

### Identification of a prognosis-related module based on WGCNA

We constructed a scale-free signed network based on the soft threshold. A total of six nongray modules were generated for further analysis by hierarchical clustering of adjacency-based dissimilarity. The gene dendrograms are shown in Fig. S1A. A topological overlap heatmap was drawn with 400 randomly selected DEGs that showed a high degree of topological overlap of genes per module (Fig. S1B). The heatmap revealed the eigengene adjacency of six co-expression modules (Fig. S1C). To identify the genes associated with certain clinical traits, we analyzed the relevance between modules and the clinical parameters of GC. As shown in Fig. S1D, the blue-green module was significantly associated with histologic grade, number of positive lymph nodes, and pathological T category. Therefore, we focused on the blue-green module as the most relevant to the OS of GC patients.

### Construction of lncRNA and mRNA prognostic signatures

To further explore the prognostic value of all genes in the blue-green module, univariate Cox regression analysis of OS was performed. The results indicated that 78 genes encoding 16 lncRNAs and 62 mRNAs were correlated with the prognosis of GC (Fig. S2). These genes were selected for subsequent analyses and construction of prognostic signatures

**Figure 4.**
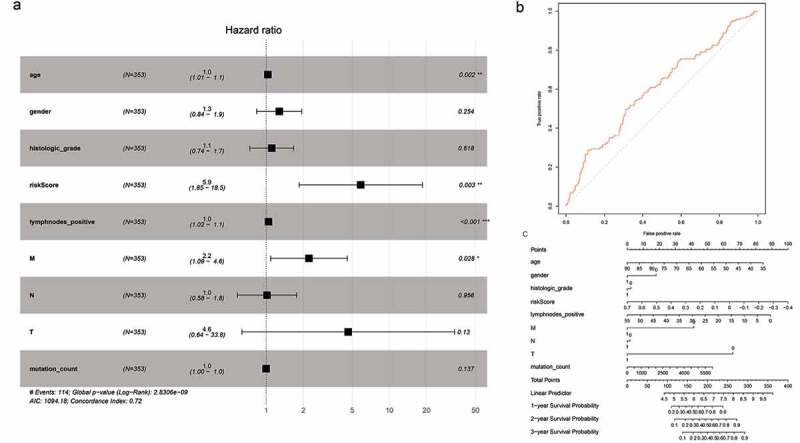
Cox proportional hazards regression analysis of lncRNAs. (a) Forest plot of risk factors. (b) The AUC for risk score was calculated according to the ROC curve. (c) Nomogram of OS prediction in GC

To minimize the risk of overfitting the prognostic signature, we performed LASSO regression analysis with tenfold cross-validation on these 16 lncRNAs. Using multivariate Cox regression analysis, we built a 5-lncRNA prognostic risk signature (Fig. S3A-C). Risk score = e (RP11-248N22.1 * 0.007573 + FGF10-AS1* 0.0005717+ RP11-21C4.1*0.003929 + RP11-963H4.6 * 0.01052 + LINC01697 * 0.02340). The patients were divided into high-risk (n = 176) and low-risk (n = 177) groups on the basis of the median risk score. We subsequently performed univariate Cox regression analysis to identify prognostic factors. Eventually, age, risk score, number of positive lymph nodes, and pathologic M category were shown to be significantly correlated with OS (Figure 4(a)). The AUC-ROC of the 5-lncRNA signature was 0.605, with a high predictive value for survival (Figure 4(b)). The results of the multivariate Cox regression analysis were visualized by drawing a nomogram (Figure 4(c))

**Figure 5.**
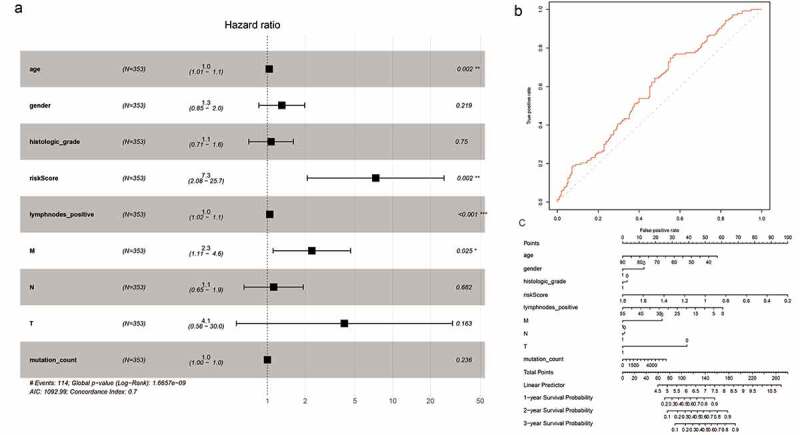
Cox proportional hazard regression analysis of mRNAs. (a) Forest plot of risk factors. (b) The AUC for the risk score was calculated according to the ROC curve. (c) Nomogram of OS prediction in GC

The analysis of 62 mRNAs was performed as described previously. Finally, a 4-mRNA prognostic risk signature was constructed (Fig. S4A-C). Risk score = e (APOD * 0.02833 + STK32A * 0.04637 + SVEP1 * 0.01973 + NPTX1 * 0.005390). On the basis of the median risk score, the patients were divided into high-risk (N = 176) and low-risk (N = 177) groups (Figure 5(a)). Univariate Cox regression analysis showed that age, risk score, number of positive lymph nodes, and pathologic M category were significantly correlated with OS (Figure 5(a)). The AUC-ROC value of the prognostic signature was 0.599 (Figure 5(b)). The results of the multivariate Cox regression analysis were visualized by drawing a nomogram (Figure 5(c)).

## *Identification of the* RP11-21C4.1/SVEP1 *gene pair as a prognostic biomarker in GC*

To better understand the effects of the genes in the prognostic models, we compared the respective risk scores among different clinical traits. As shown in Fig. S5, the risk score of the 5-lncRNA signature was significantly related to histologic grade, gender, and age, while the risk score of the 4-mRNA signature was significantly related to histologic grade, pathologic T category, tumor grade, and age. The results demonstrated that the mRNA and lncRNA prognostic models showed good applications in terms of age and histologic grade. The risk score of the 5-lncRNA signature showed no significant difference in relation to the pathologic T category. However, the two prognostic models showed a high risk score in the group with a high degree of primary tumor invasion, suggesting that these identified genes could be prognostic biomarkers in GC.

**Figure 6.**
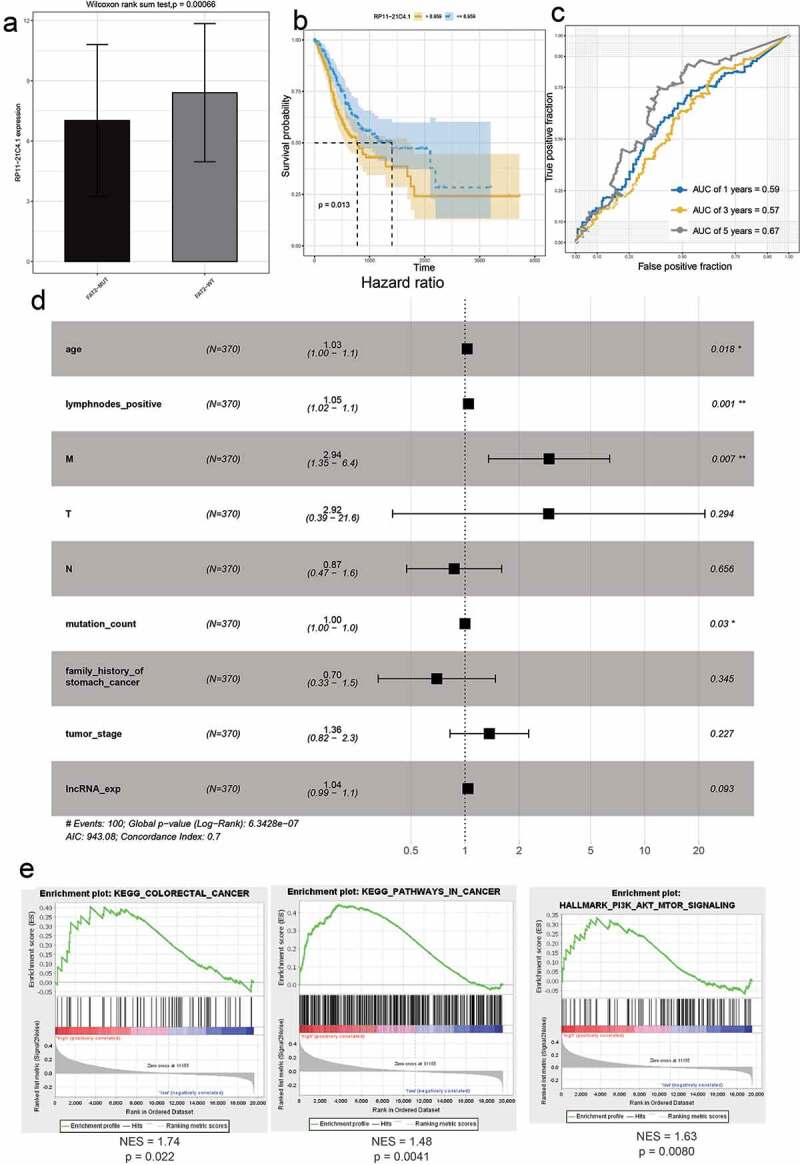
The effect of RP11-21C4.1 on the prognosis of GC. (a) The expression value of RP11-21C4.1 in FAT2-mutant and FAT2-wildtype GC. (b) Kaplan–Meier curves for the OS of GC patients in the high- and low-RP11-21C4.1 groups. (c) The AUC for RP11-21C4.1 was calculated according to the ROC curve. (d) Multivariable Cox regression analysis. (e) Cancer gene enrichment analysis based on the state of RP11-21C4.1 expression

**Figure 7.**
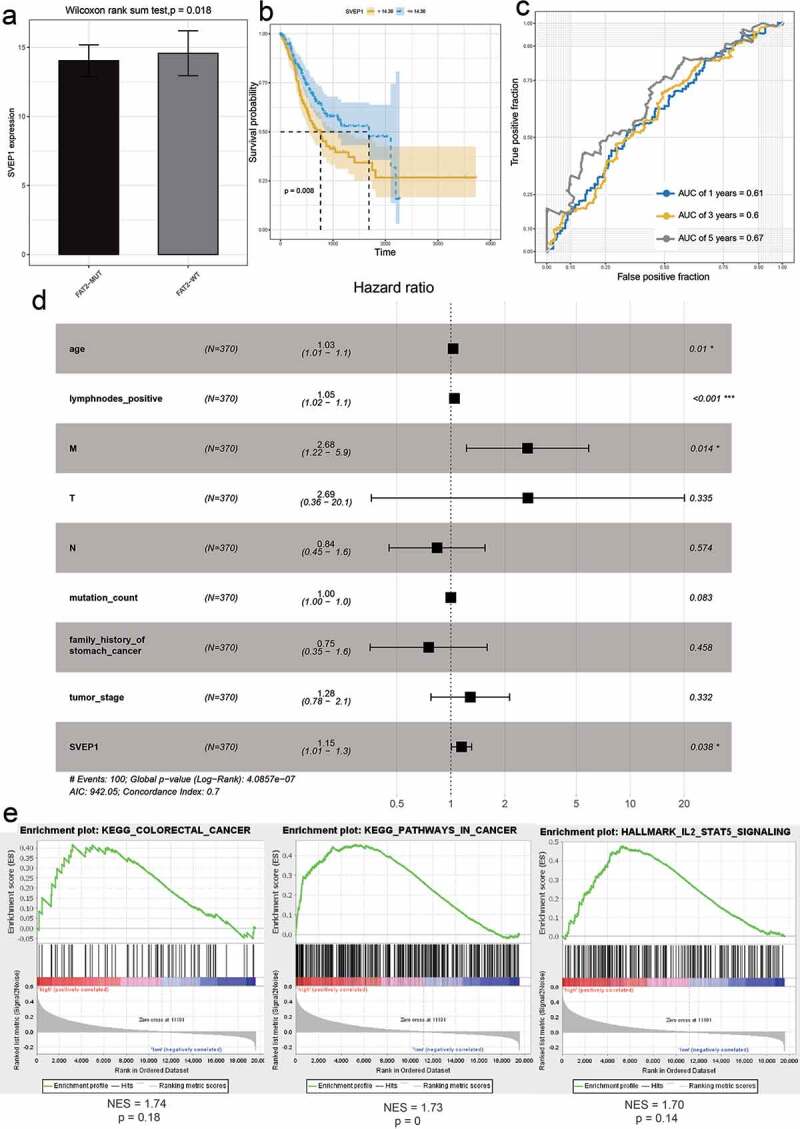
The effect of SVEP1 on the prognosis of GC. (a) The expression value of SVEP1 in FAT2-mutant and FAT2-wildtype GC. (b) Kaplan–Meier curves for the OS of GC patients in the SVEP1 high- and low-expression groups. (c) The AUC for SVEP1 was calculated according to the ROC curve. (d) Multivariable Cox regression analysis. (e) Gene enrichment analysis based on the state of SVEP1 expression

LncRNAs drive many important cancer phenotypes through their interactions with mRNA, and they have been described to play roles in the control of mRNA stability, splicing, and translation [15]. Therefore, we analyzed the correlation between mRNAs and lncRNAs in prognostic models. *RP11-21C4.1/SVEP1* was identified as the most relevant gene pair (r = 0.54, P < 0.05) (Fig. S6). We then studied the clinical significance of *RP11-21C4.1* and *SVEP1* in GC. As expected, *RP11-21C4.1* and *SVEP1* were coordinately downregulated in FAT2-mutant samples (Figure 6(a); Figure 7(a)). These results suggest that *RP11-21C4.1* may play a role in the control of *SVEP1* expression during the development of GC. High expression of *RP11-21C4.1* or *SVEP1* was associated with a poor OS (Figure 6(b); Figure 7(b)). The AUC-ROC of *RP11-21C4.1* or *SVEP1* for OS was 0.67 at 5 years (Figure 6(c); Figure 7(c)), suggesting that both genes have a good predictive performance for OS. It has been reported that two-gene markers showed more sensitive and specific than single-gene markers, which provided more accurate indicator for the diagnosis of cancers. We further plotted the ROC curves of two-biomarker combinations, which suggested that AUC values were 0.69 at 5 years (Fig. S7). In terms of the prognostic value, two-biomarker combinations are superior than RP11-21C4.1 or SVEP1 gene. In the univariate Cox regression analysis, the hazard ratios (HRs) of *RP11-21C4.1* and *SVEP1* were 1.04 and 1.15, respectively (Figure 6(d); Figure 7(d)). This proved that *RP11-21C4.1* and *SVEP1* were independent prognostic factors for GC. Furthermore, the set of genes associated with cancers, including GC, was enriched in the *RP11-21C4.1* and *SVEP1* high-expression phenotype (Figure 6(e); Figure 7(e)).

### Construction of the TF-mRNA network based on candidate genes

**Figure 8.**
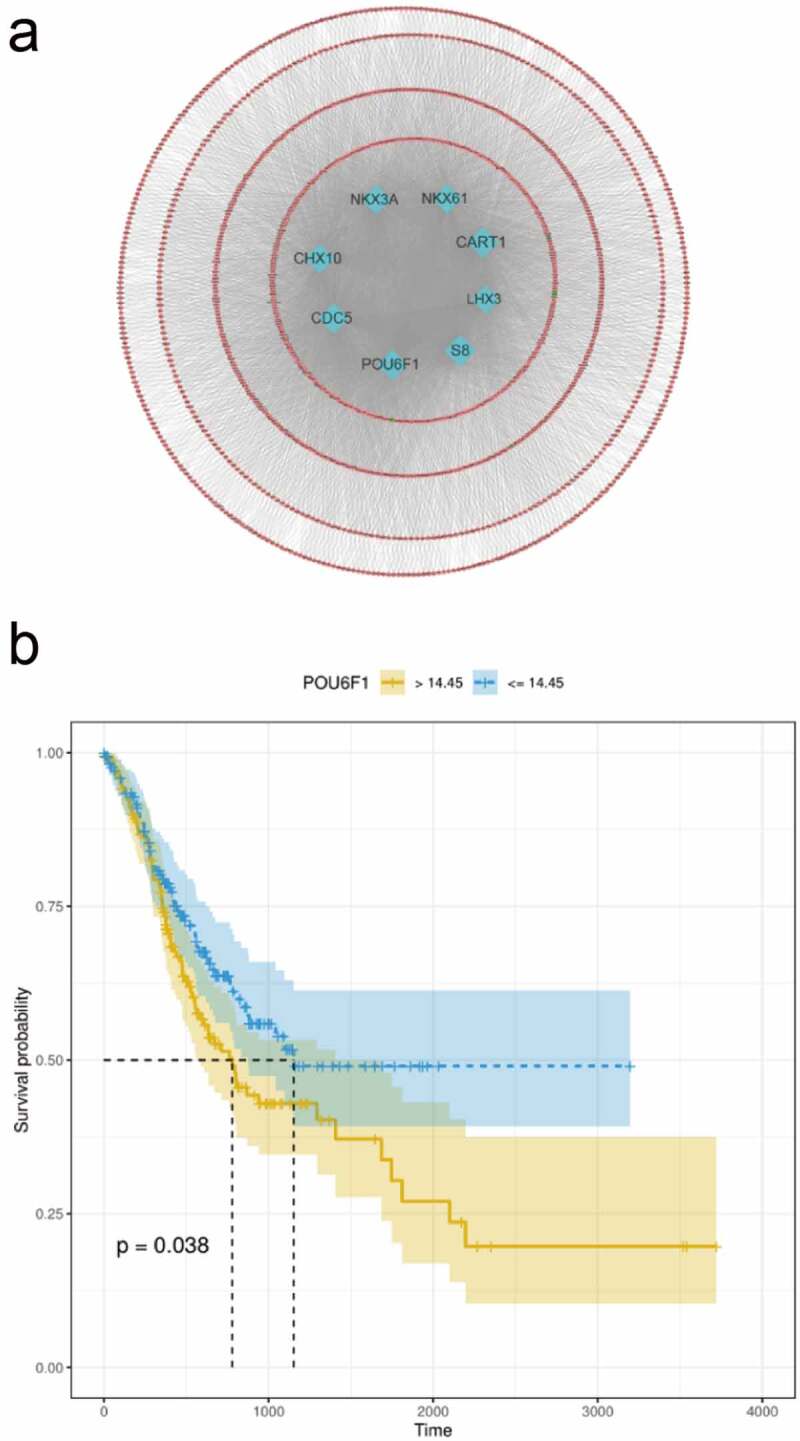
Construction of the TF-mRNA network. (a) TFs enriched for DEGs between the SVEP1 high- and low-expression groups. (b) The association between POU6F1 and the prognosis of GC

To identify the targets of the *RP11-21C4.1/SVEP1* gene pair, we constructed a TF-mRNA network. A total of 1589 DEGs were identified between *SVEP1* high-expression and *SVEP1* low-expression groups. Finally, TFs (CHX10, CDC5, POU6F1, S8, LHX3, CART1, NKX61, and NKX3A) associated with cancers were significantly enriched in DEGs. These TFs may be potential targets of *SVEP1* (Figure 8(a)). We also explored the association between the eight TFs and GC prognosis. As shown in Figure 8(b), POU6F1 was linked to GC prognosis.

## Discussion

Gastric cancer is a disease with marked heterogeneity that can result in poor response to conventional treatments, contributing to a high mortality rate and poor prognosis. Traditional methods are limited and cannot address these barriers. Thus, novel investigations must be conducted to identify new biomarkers with specific prognostic potential that can aid in personalized clinical treatment decision-making [16]. With the development of high-throughput technologies, a landscape of signatures is emerging to fulfil the vision of precision medicine for cancer. In this study, we analyzed DEmRNAs and DElncRNAs associated with FAT2 mutations, and their potential interactions, to identify the *RP11-21C4.1/SVEP1* gene pair as a prognostic signature of GC. Notably, the *RP11-21C4.1/SVEP1* prognostic signature exhibited superior predictive performance in comparison with a 4-mRNA or 5-lncRNA prognostic signature in GC. Integration of both mRNA and lncRNA into a prognostic model may enhance robustness. The AUC of the P11-21C4.1/SVEP1 signature was 0.67, while the previously published prognostic signature for GC only reached an AUC of 0.63 [17]. Additionally, in comparison with the prognostic signature of five genes built by Yang et al., our prognostic signature with two genes was more economical [17].

Mutations observed in cancer cells have different functional effects; some do not have a noticeable impact, and others can alter key functions such as oncogenic activation and tumor suppression [18]. Millions of somatic mutations in cancer genomes have been identified by genome sequencing. However, the functional effects of most somatic mutations in cancer remain unknown. Identification of driver mutations in cancer and elucidation of their oncogenic mechanisms represents a challenge in implementing precision medicine [19]. FAT2 mutations occur in many cancers, including GC. To date, the effect of these mutations on the prognosis of GC has not been studied. In this study, we found that patients with FAT2 mutations showed better prognosis, suggesting that these mutations play a role in tumor suppression in GC. To better understand the functional effects of FAT2 mutations in GC, we explored the DEmRNAs and DElncRNAs based on the status of FAT2 mutations, and found that these DEGs were mainly enriched in the intracellular material transport and signal transduction-related biological processes and pathways. FAT2 is a member of the cadherin superfamily and acts as a transmembrane receptor for Hippo signaling [6,20], which is consistent with our findings. Dysregulation of the Hippo pathway signaling is frequently observed in GC, contributing to tumor progression [21]. This might be one of the mechanisms by which FAT2 mutations affect the prognosis of GC.

Numerous studies have found some genomic mutations in regions that do not encode proteins but are often transcribed into lncRNAs [22]. The long noncoding transcriptome is often dysregulated in cancers, and some of these dysregulations are associated with malignant transformation [23]. We identified the prognostic DElncRNAs associated with FAT2 mutations and constructed a 5-lncRNA prognostic signature of GC (RP11-248N22.1, FGF10-AS1, RP11-21C4.1, LINC01697, and RP11-248N22.1). One study showed that FGF10-AS1 was associated with the prognosis of triple-negative breast cancer patients, which is consistent with our research [24]. The lncRNA FGF10‐AS1 may play a role in the development of many cancers. In addition, the lncRNA LINC01697 also showed good predictive performance in the diagnosis and prognosis of lung squamous cell carcinoma or oral squamous cell carcinoma [25,26]. The other lncRNAs in this model have not been reported in cancer and require further research.

The presence of an intricate interplay between protein-coding messenger RNAs and non-coding RNAs is becoming increasingly evident. A thorough understanding of this RNA crosstalk will contribute to a deeper understanding of cancer biology [27]. In this study, we further analyzed the DEmRNAs associated with FAT2 mutations and constructed a 4-mRNA prognostic signature of GC (APOD, STK32A, SVEP1, NPTX1). Many studies have demonstrated that APOD expression is correlated with various tumors, such as breast cancer and colorectal cancer, and is significantly associated with GC risk assessment [28–30]. STK32A participates in cellular homeostasis, cell-cycle modulation, and phosphorylation of transcription factors, and can modulate the invasion and metastasis of non-small cell lung cancer [31]. NPTX1 was also found to be aberrantly expressed in multiple cancers and plays a crucial role in promoting metastasis in GC [32]. These studies further confirmed the accuracy of our results.

To better understand RNA crosstalk, we analyzed the correlation between lncRNAs and mRNAs, and identified the most relevant *RP11-21C4.1/SVEP1* gene pair. Previous studies have described that a significant proportion of lncRNAs originate from the divergent transcription of nearby protein-coding genes. These divergent lncRNA/mRNA gene pairs exhibit genomic juxtaposition and co-expression in transcription [33]. The evidence described in prior research suggests that divergent lncRNAs can regulate the transcription of nearby genes. The biological function of these lncRNAs may be inferred from the role of their nearby protein-coding genes [34]. Our results showed that *RP11-21C4.1* and *SVEP1* were coordinately downregulated in FAT2-mutant samples, suggesting that *RP11-21C4.1* may act as a divergent lncRNA to regulate the transcription of *SVEP1*. To date, research on *RP11-21C4.1* has not been reported. *RP11-21C4.1* is a pseudogene, and there is substantial evidence that pseudogenes play important roles in the pathogenesis of cancer by regulating their parental or unrelated genes [35]. Many studies have shown that pseudogenes associated with *RP11* are correlated with the development of many cancers, including GC [36]. The effects of *RP11-21C4.1* on GC require further study. *SVEP1* is involved in the regulation of intercellular adhesion, and its aberrant expression can induce hepatocellular carcinoma proliferation and metastasis [37]. Further investigation is required to elucidate the regulation of *SVEP1* expression in GC.

## Conclusion

In this study, we constructed an *RP11-21C4.1/SVEP1* gene pair prognostic signature associated with FAT2 mutations by using integrative genomics analysis. Our findings will improve our understanding of the potential biological functions of FAT2 mutations affecting the prognosis of GC.

## Supplementary Material

Supplemental Material

## Data Availability

The data of this study are available in the TCGA (https://portal.gdc.cancer.gov).
