# Supplementary material for: Identification of the RP11-21C4.1/SVEP1 gene pair associated with FAT2 mutations as a potential biomarker in gastric cancer
Source: Bioengineered. 2021 Jul 24;12(1):4361–73. doi: 10.1080/21655979.2021.1953211 (PMC8806586; doi:10.1080/21655979.2021.1953211)
Supplement: Supplemental Material [file KBIE_A_1953211_SM1570.zip › supplementary/Supplemental Figure Legendsclean.docx]

**Fig.S1**. WGCNA and identification of the modules associated with the prognosis of GC. (A) Module hierarchical clustering dendrogram. (B)  Network heatmap based on topological overlap. (C) The heatmap of relationship among modules. (D) Module&clinical trait relationships

**Fig.S2**. Kaplan-Meier curves for the OS. (A-C) OS analysis for lncRNAs in blue-green module. (D-E) OS analysis for mRNAs in the blue-green module.

**Fig.S3**. LASSO analysis for screening lncRNAs in prognostic risk models. (A) HR forest map of the top 30 lncRNAs in the blue-green module. (B) LASSO analysis to determine penalty parameter (λ) for the model. (C) The coefficients of lncRNAs included in this model.

**Fig.S4**. LASSO analysis for screening mRNAs in prognostic risk models. (A) HR forest map of the top 30 mRNAs in the blue-green module. (B) LASSO analysis to determine penalty parameter (λ) for the model. (C) The coefficients of mRNAs included in this model.

**Fig.S5**. Association between the risk scores and clinical characteristics. (A) Association between risk score in the lncRNA prognostic model and different clinical features. (B) Association between risk score in the mRNA prognostic model and different clinical features.

**Fig.S6**. [Correlation](javascript:;) [analysis](javascript:;) between RP11-21C4.1 and SVEP1.

**Fig.S7.** The AUC for RP11-21C4.1 and SVEP1 combinations was calculated according to the ROC curve.
