# Supplementary figures and images for: Identification of the RP11-21C4.1/SVEP1 gene pair associated with FAT2 mutations as a potential biomarker in gastric cancer
Source: Bioengineered. 2021 Jul 24;12(1):4361–73. doi: 10.1080/21655979.2021.1953211 (PMC8806586; doi:10.1080/21655979.2021.1953211)

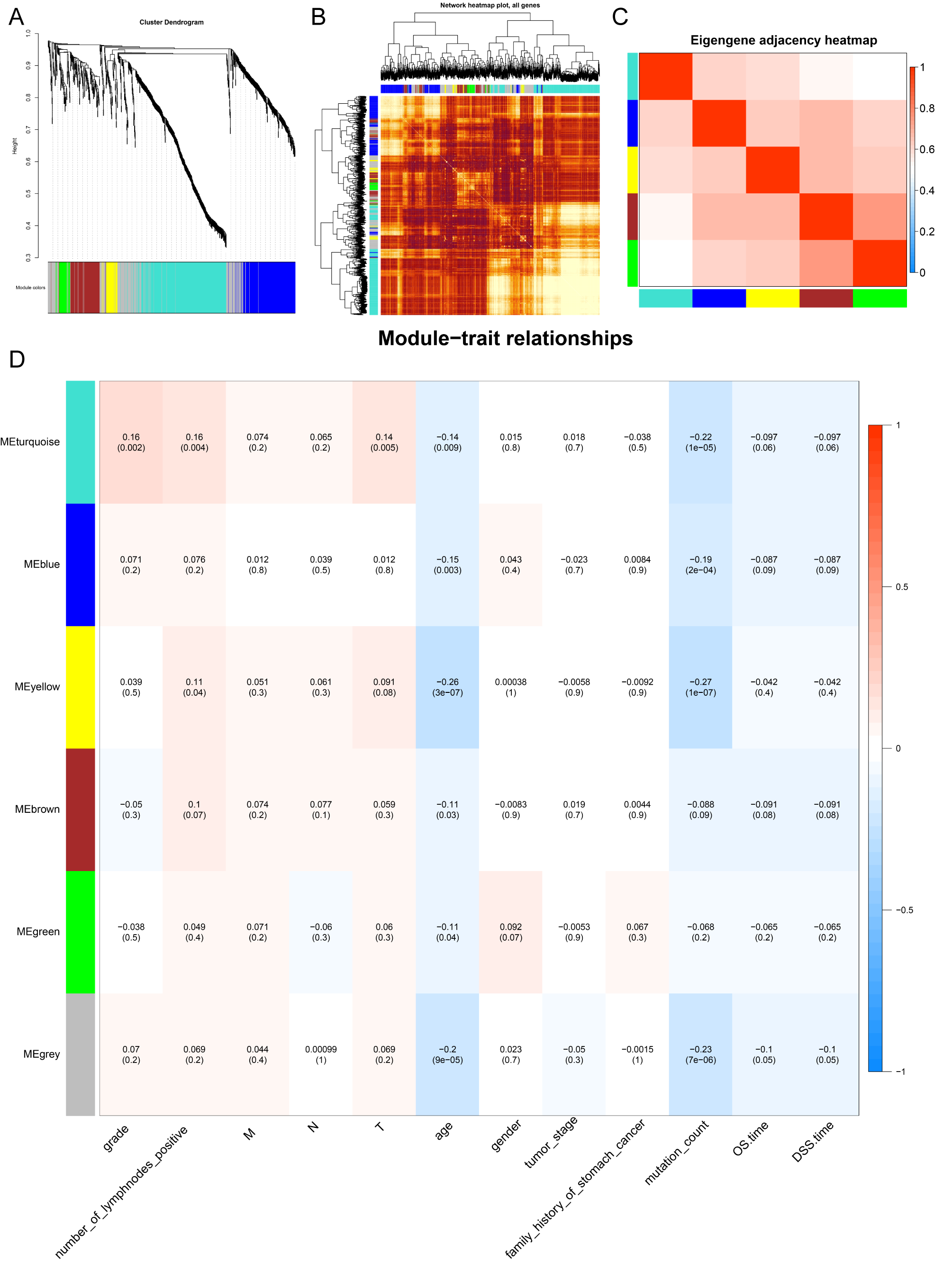

Supplement: Supplemental Material [file KBIE_A_1953211_SM1570.zip › supplementary/SupplementalFigS1.tif]

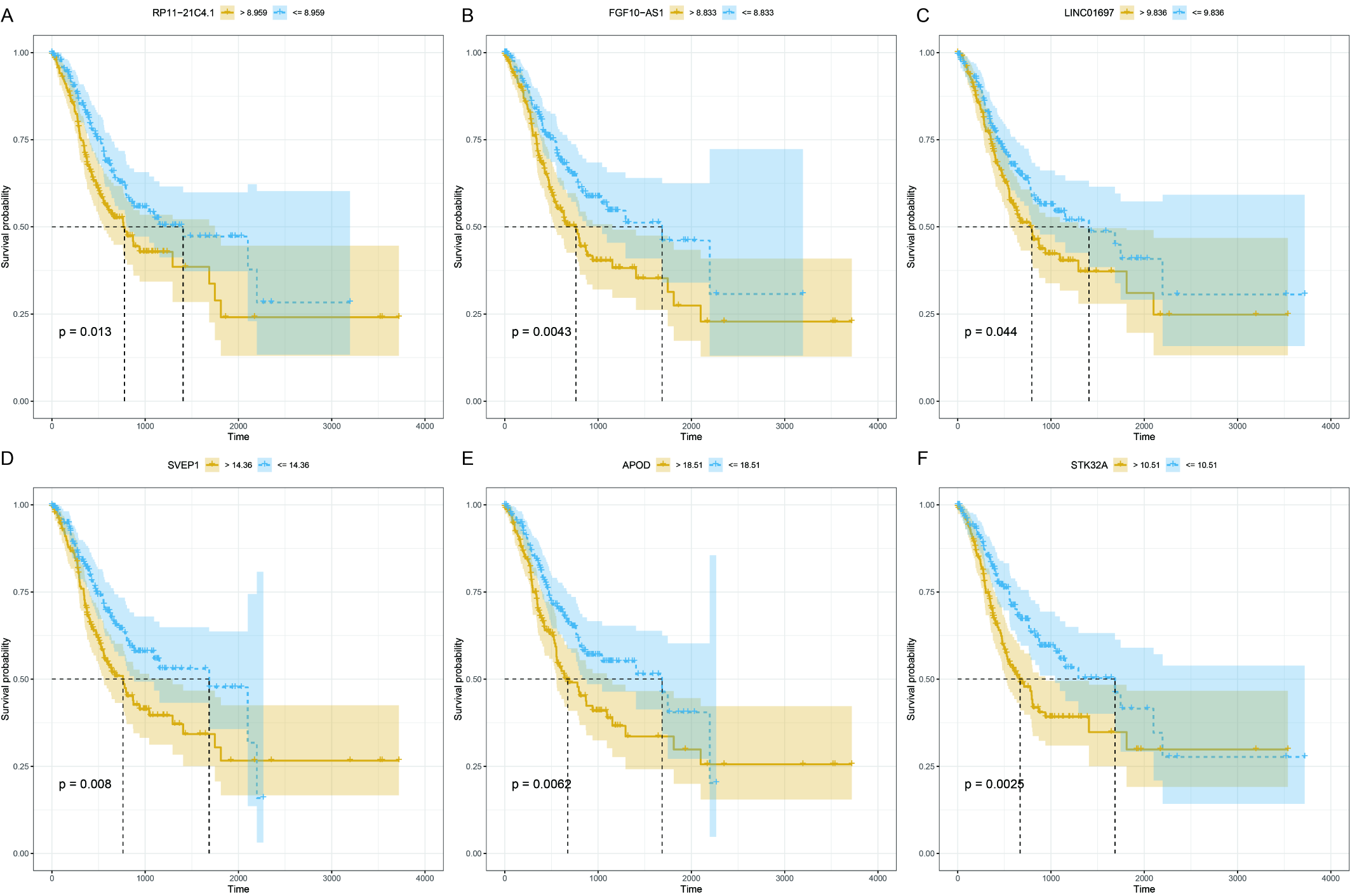

Supplement: Supplemental Material [file KBIE_A_1953211_SM1570.zip › supplementary/SupplementalFigS2.tif]

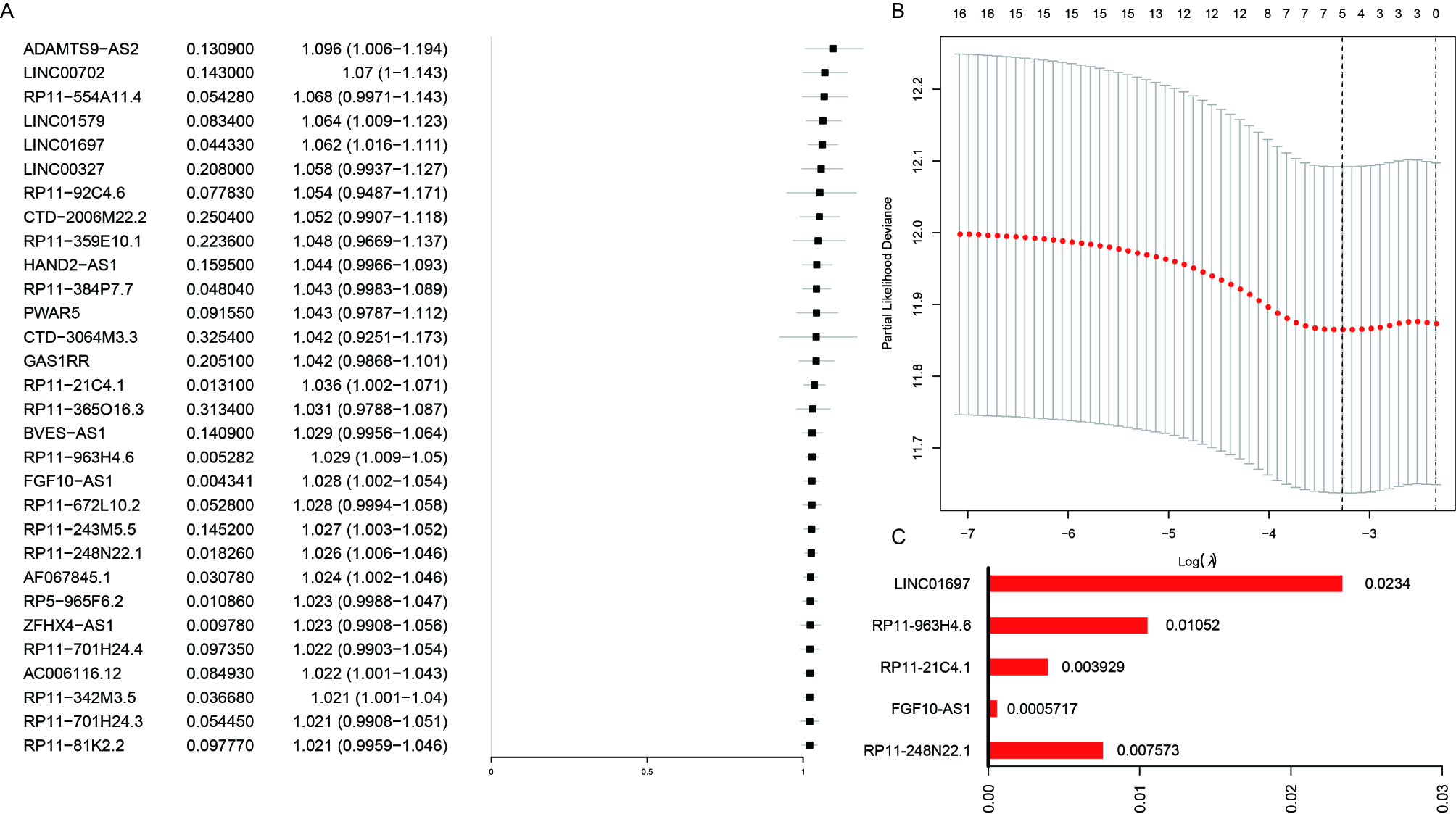

Supplement: Supplemental Material [file KBIE_A_1953211_SM1570.zip › supplementary/SupplementalFigS3.tif]

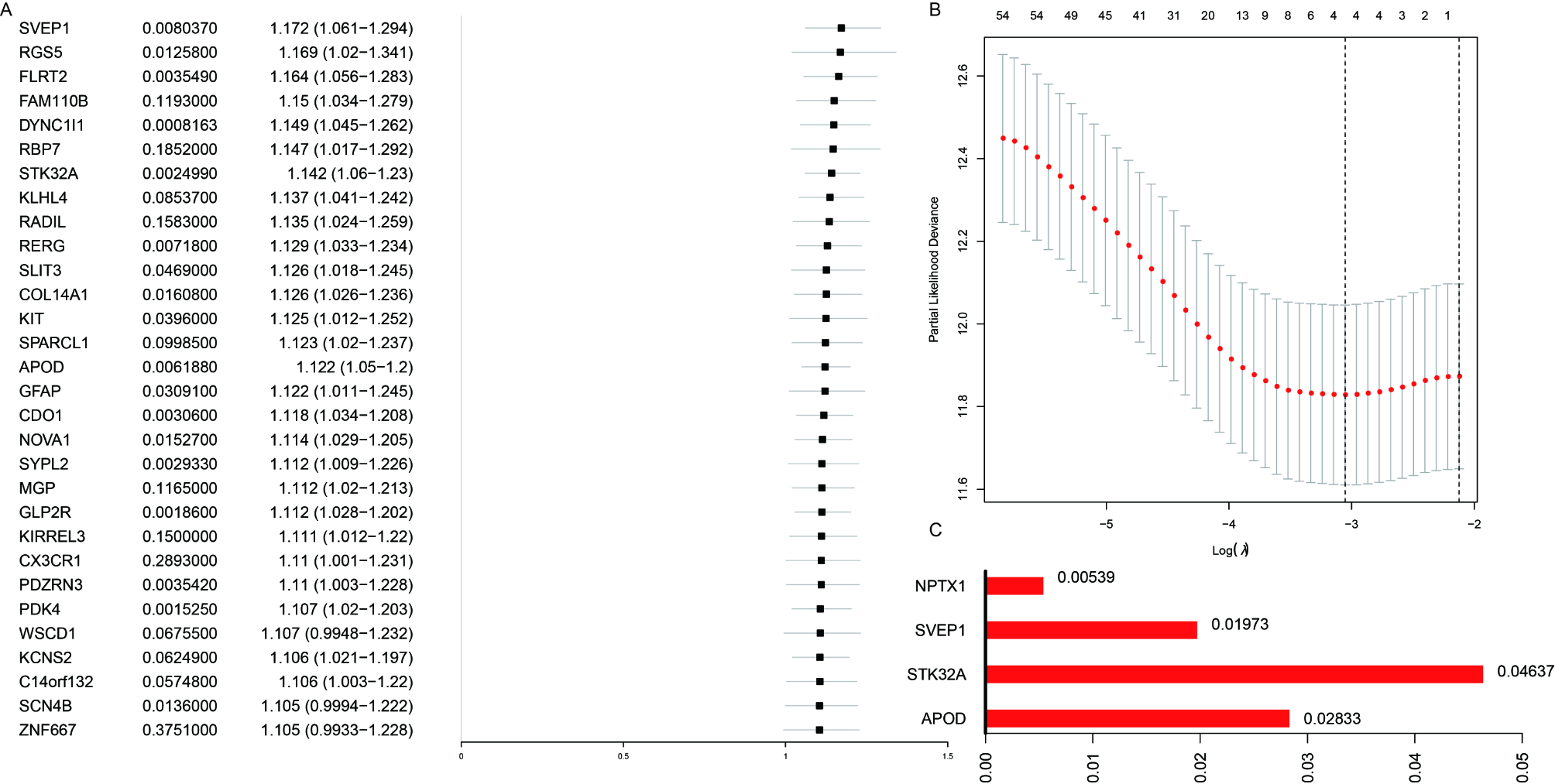

Supplement: Supplemental Material [file KBIE_A_1953211_SM1570.zip › supplementary/SupplementalFigS4.tif]

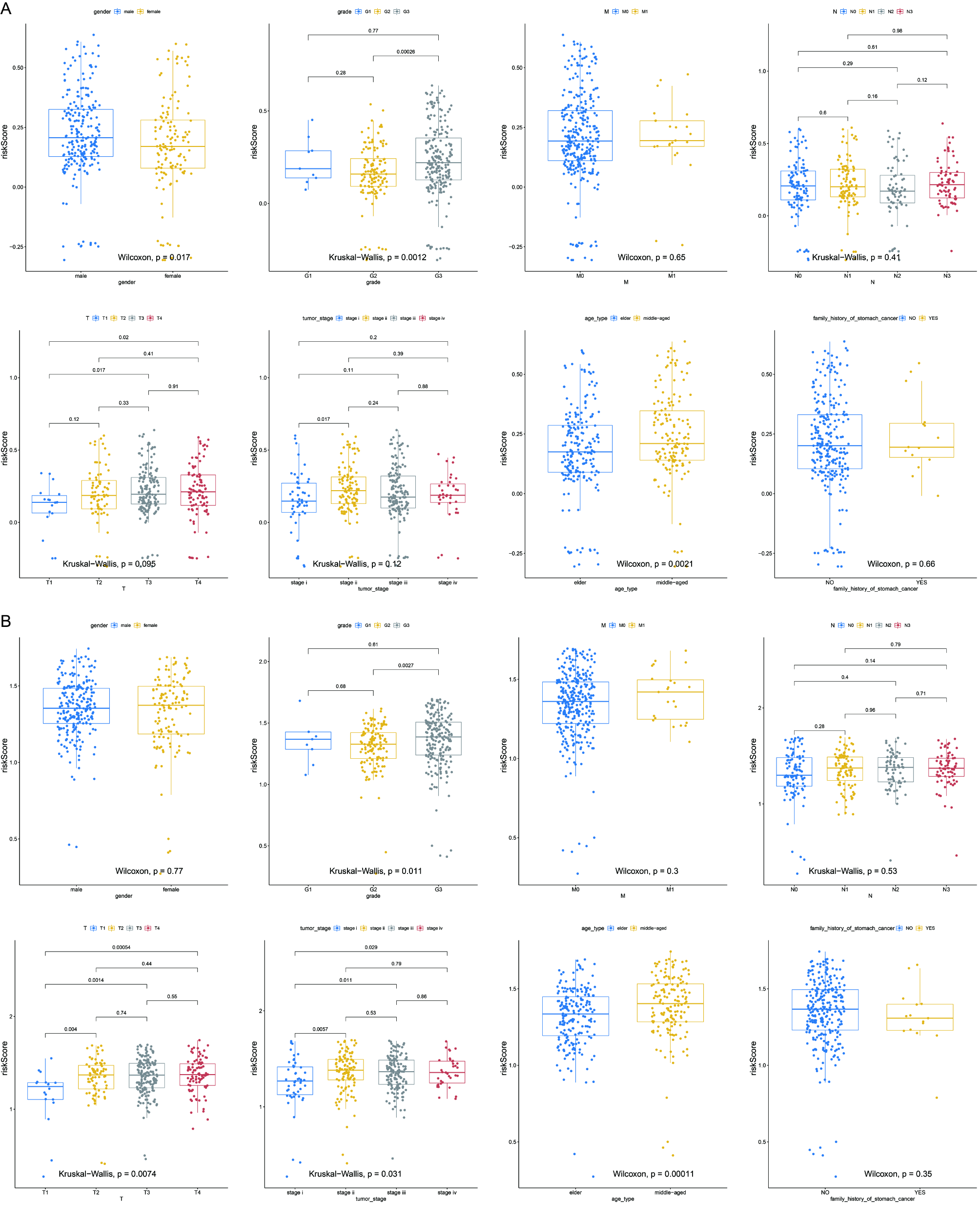

Supplement: Supplemental Material [file KBIE_A_1953211_SM1570.zip › supplementary/SupplementalFigS5.tif]

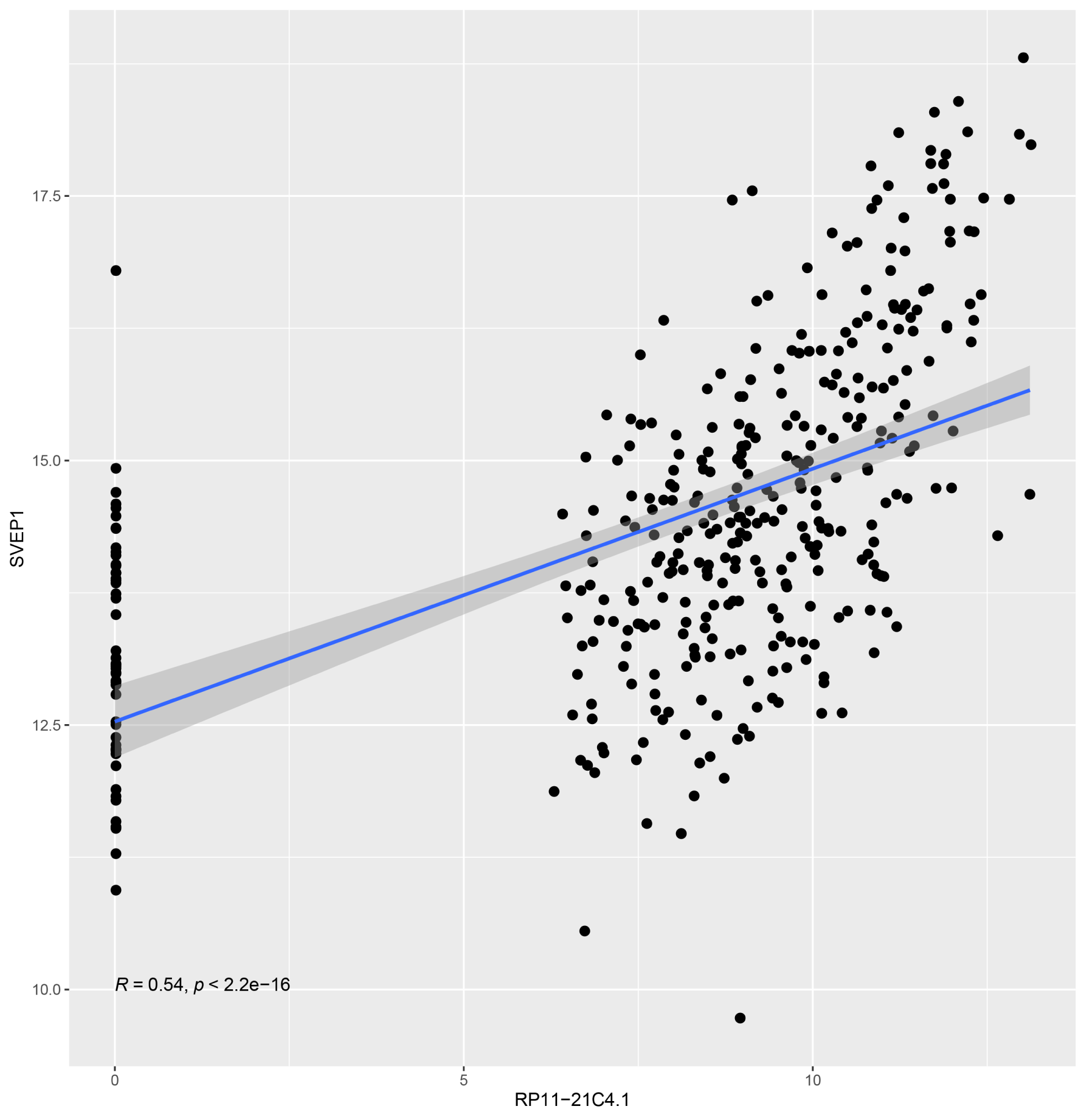

Supplement: Supplemental Material [file KBIE_A_1953211_SM1570.zip › supplementary/SupplementalFigS6.tif.yjw21uf.partial]

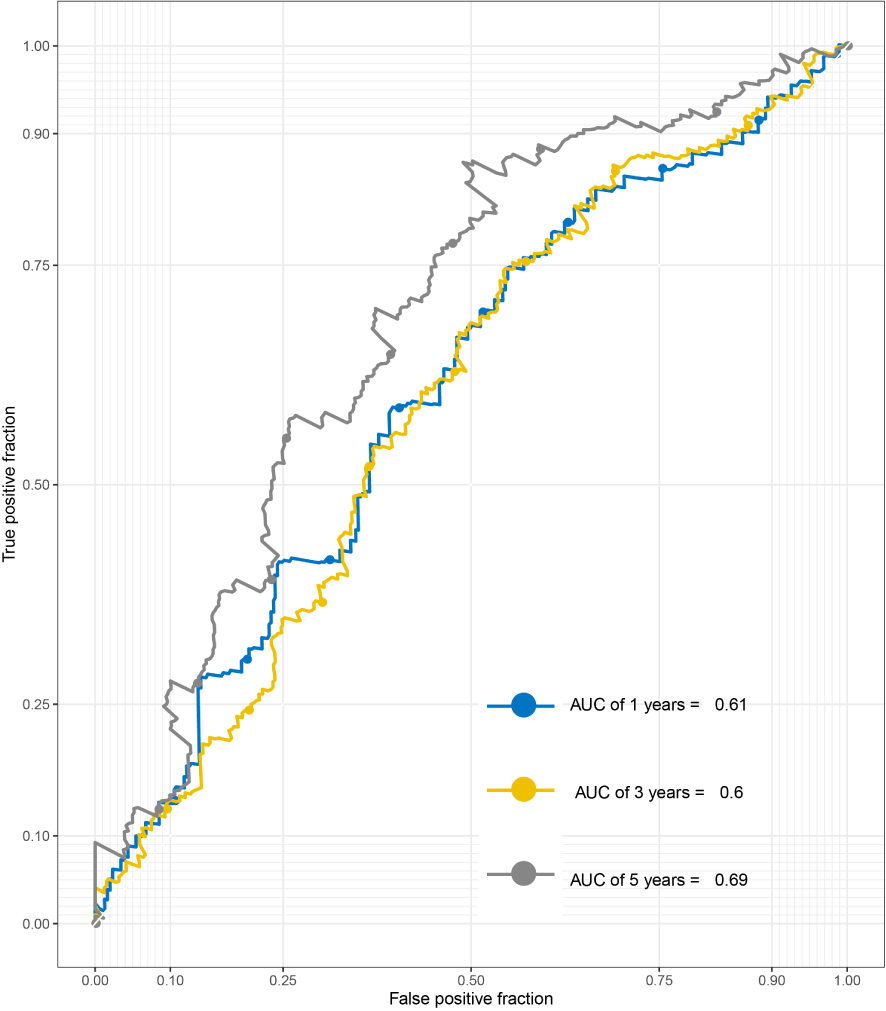

Supplement: Supplemental Material [file KBIE_A_1953211_SM1570.zip › supplementary/SupplementalFigS7.tif]
